# The role of the river in the functioning of marginal fen: a case study from the Biebrza Wetlands

**DOI:** 10.7717/peerj.13418

**Published:** 2022-06-22

**Authors:** Maria Grodzka-Łukaszewska, Grzegorz Sinicyn, Mateusz Grygoruk, Dorota Mirosław-Świątek, Ignacy Kardel, Tomasz Okruszko

**Affiliations:** 1 Faculty of Building Services, Hydro and Environmental Engineering, Warsaw University of Technology, Warsaw, Poland; 2 Institute of Environmental Engineering, Warsaw University of Life Sciences-SGGW, Warsaw, Poland

**Keywords:** River-fen relations, Marginal fens, Fen functioning, River-fen model, Fen protection

## Abstract

**Study region:**

The area of interest is the Upper Biebrza Valley, located in NE Poland.

**Study focus:**

We examined water exchange at the river-fen interface in a near-natural wetland system using the combined field research-modeling approach. The authors chose the Biebrza River as the research object: it is a specific case of fen marginal valley rivers, and it flows in the peat layer without direct connection to the mineral soil layer. Our case study introduces two new aspects not yet considered in the scientific literature: (1) the riparian aquifer is fen and (2) the river has no direct contact with the mineral layer. The following research questions were investigated: What is the role of the river in feeding and draining a fen? Which drainage paths are important for water exchange in a near-natural river-fen system? How important are the morphological settings for the river-fen relations? We applied a systematic hydrological research approach based on field measurements and observations of the river and surrounding fen hydrological characteristics, as well as on the modelling results.

**New hydrological insights for the region:**

We demonstrated that morphological settings have a significant influence on river-fen relations. We also demonstrated that due to the undeniable need to introduce increased protection and restoration of marginal fens, we may focus on river status in narrow valleys; however, in the wide valleys, the limitation of the drainage layer by decreasing the intensity of evapotranspiration is more promising. We propose to distinguish zones in the fen river valley to include them when proposing protection or conservation plans.

## Introduction

Mires and peatlands (which we consider as drained mires in this article) are recognized as ecosystems of wide significance for global biodiversity. This ecosystem represents one of the largest natural terrestrial carbon reservoirs (Leifeld & Menichetti, 2018; Yu et al., 2011). For example, northern and central European lowlands used to have well-developed fen vegetation in river valleys and floodplains. However, in recent decades, fen vegetation, even in nature reserves, has deteriorated due to increasing anthropopressure. Hydrological interferences such as abstraction of groundwater for drinking water supply, drainage of agricultural land, and other aspects of agricultural intensification such as high fertilization have had a marked negative effect on the amount and composition of water in fen areas (Verhoeven & Toth, 1995; Koerselman, Bakker & Blom, 1990; Wassen et al., 1990; Schot, 1991; Van Diggelen et al., 1991; Günther et al., 2020). If the remaining vegetation is to be preserved or the fen vegetation is to be restored in these areas, we must maintain proper hydrological conditions. This means that we should thoroughly understand the mechanisms of recharging peat with water. There is no doubt that wetlands should be protected by maintaining an appropriate level of groundwater in them; per Kreyling et al. (2021), rewetting does not return drained fen peatlands to their old selves.

There are three main sources of water that support the growth of peat under oxygen lack and waterlogged conditions. For fens, the main one is groundwater supported by precipitation. In the case of bogs, rain and snowmelt create waterlogged conditions (Okruszko & Kiczko, 2008). Inundation coming from rivers creates a peat-forming habitat in very particular cases of long-lasting inundation (Keizer et al., 2016). Due to the difference in chemical properties of the water coming from the mentioned sources, the hydrology of mires or peatlands not only clarifies the characteristics of the ecosystem, but also determines the best ways to protect the mires and restitution of the peatlands. In the case of fens, rivers play the role of a recipient of water throughflow in the peat body. When groundwater recharge is considered, this appears to be the most constant inflow, independent of current weather and climatic conditions. However, groundwater in peat is generally conceptualized as being at least partially isolated from the aquifer below (Regan et al., 2019). There is also uncertainty about the importance of runoff from valleys in relation to groundwater and the importance of retaining rainwater or floods (Bootsma & Wassen, 1996). These estimates are impossible to obtain by measurements, so the most appropriate way is to use mathematical modeling in this case. The utility of groundwater models as a tool for wetlands water flow analysis has been well established (*e.g.*, Batelaan & Kuntohadi, 2002; Grygoruk et al., 2011; Rajabi, Ataie-Ashtiani & Simmons, 2018; Refsgaard et al., 2010). Full recognition of the mechanisms of water flow between the peat and the aquifer will provide better management by forecasting this water exchange in climate change scenarios. The literature studies concentrate on the exchange of water between a river and a peat, nevertheless these are cases where it has direct contact with the aquifer (see, for example, Dahl et al., 2007). Thus, there is a lack of comprehensive studies on the interaction of the peat layer with the river that flows through it without touching the mineral deposits aquifer directly. Therefore, these studies outlining a new, very important direction of scientific research . However, it is clear that they form special case for water habitas and ecosystems (Grygoruk et al., 2021). Our case study introduces two new aspects not yet considered in the scientific literature: (1) the riparian aquifer is fen, (2) the river has no direct contact with the mineral layer. It is a situation where the river exchanges water only with the peat layer, it is not recharged directly from the mineral layer.

This study covers several research questions to increase our knowledge of the hydrological functioning of river marginal fens. First, what is the role of the river in feeding and draining a fen? Which drainage routes remain responsible for water exchange in a near-natural river fen system? Finally, how important are the morphological settings for the river-fen relations? To answer these questions, we applied a systematic hydrological research approach based on field measurements and modelling results.

## Materials & Methods

### Study area

**Figure 1:**
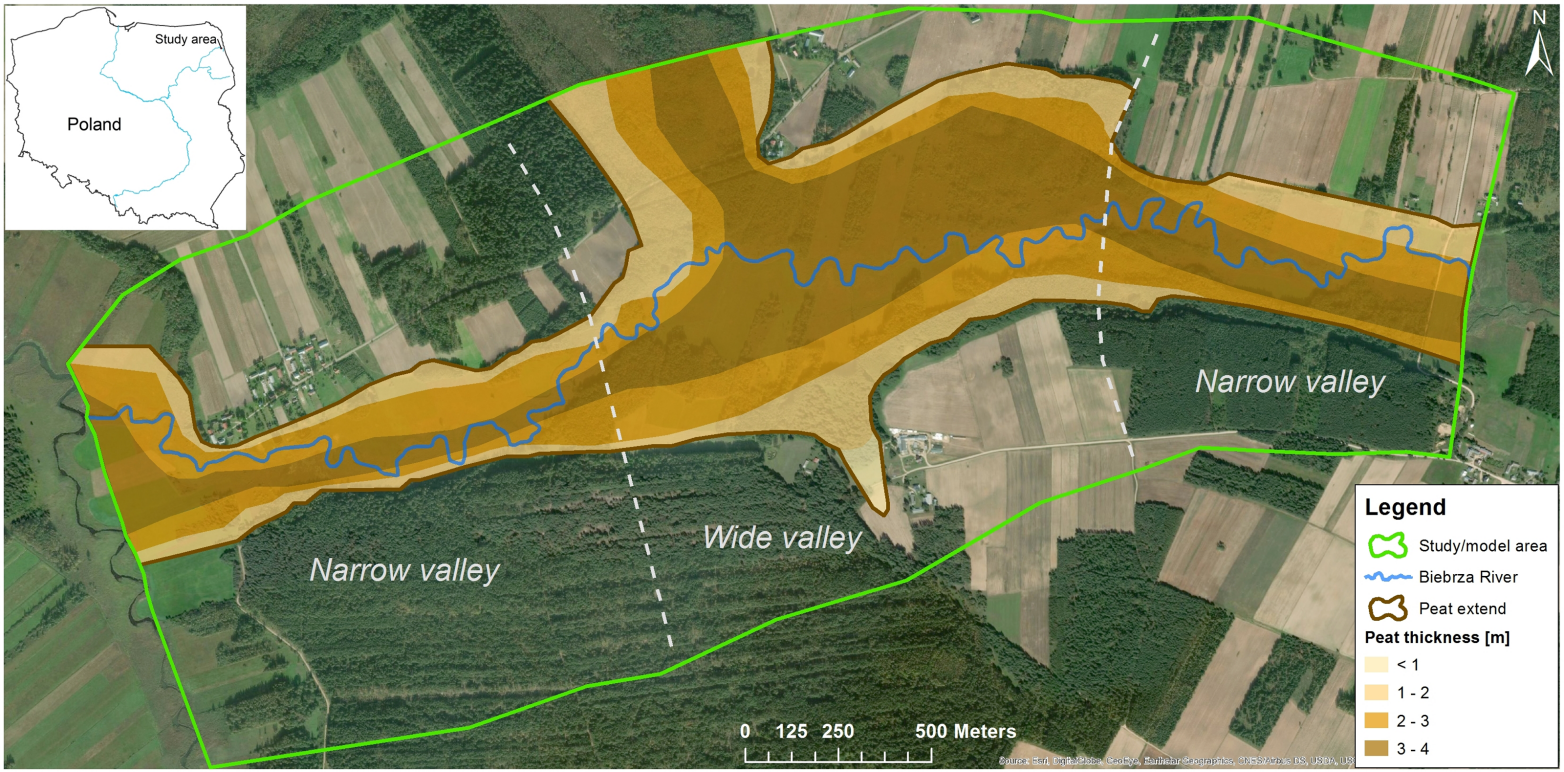
Study area.

The area of interest, the Upper Biebrza Valley, is located in NE Poland (Fig. 1). The Biebrza river is not regulated; the valley is not reclaimed and still houses marsh, fen, and grassland vegetation; large scale human interferences in hydrological system are absent. Since 1992, it has been protected as a national park (https://www.biebrza.org.pl), the protection includes a ban on river dredging and water vegetation removal. As the hydrological feeding mechanism of the Biebrza Wetlands is undisturbed, to a large extent, hydroecological studies have been conducted here for a number of years and have covered a range of the research spectrum, *e.g.*, flooding mechanism of riparian wetlands (Chormański et al., 2011; Keizer et al., 2014; Ignar et al., 2011), functioning of throughflow fens (Wassen et al., 1990), groundwater—surface water interaction (Anibas et al., 2012), changes in wetland evapotranspiration (Grygoruk, Batelaan & Miroslaw-Swiatek, 2014), physiographic factors (Jaros, 2012) and groundwater recharge (Van Loon et al., 2009; Van Loon et al., 2009b). In the study of Wassen et al. (2006), the area was called an important place of reference study in European wetland protection.

**Figure 2:**
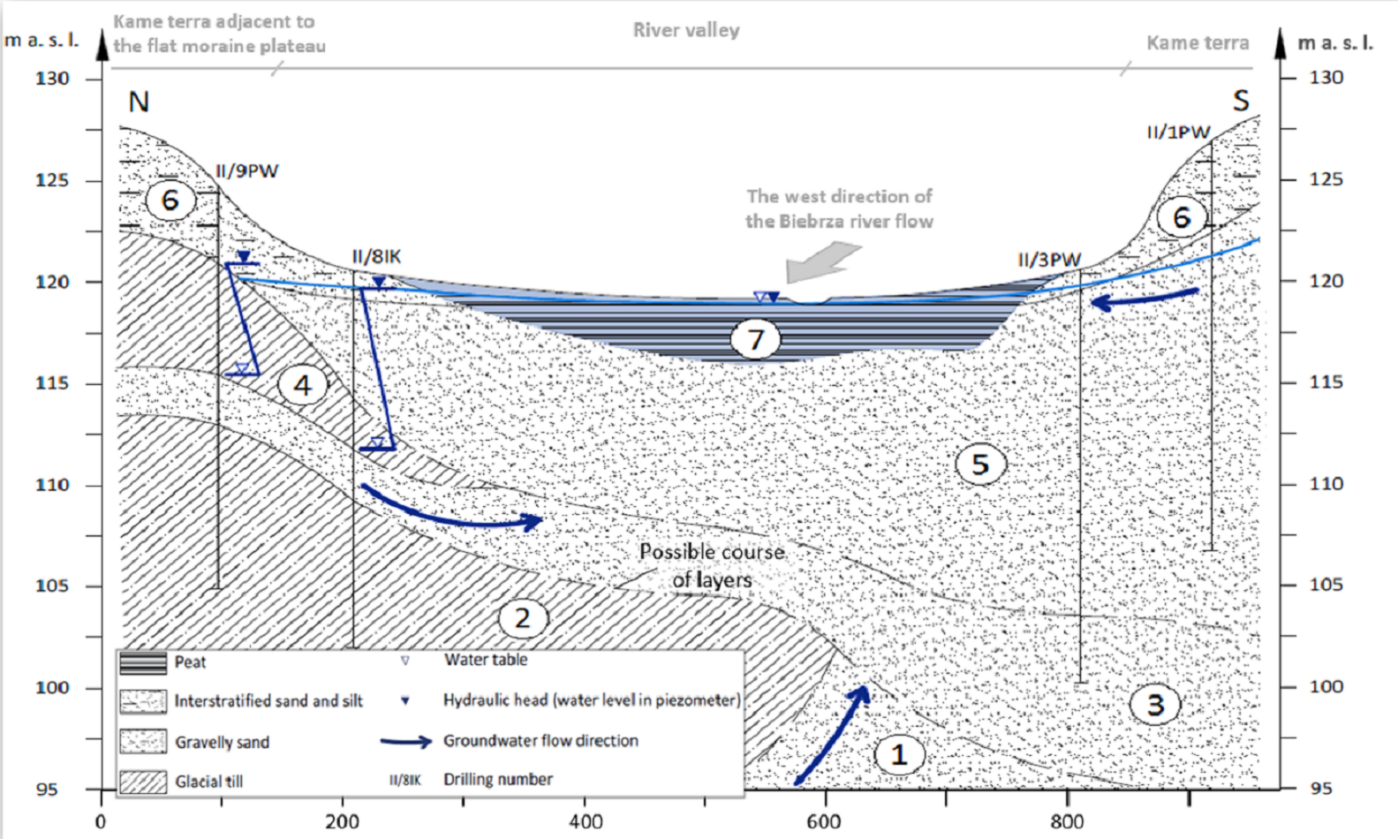
Geological cross-section through the Upper Biebrza ice-marginal valley (Vistula river catchment, NE Poland). Stratigraphy and genetic type of deposits: 1—Saalian (Odra) fluvioglacial sediments; 2—Saalian (Odra) glacial sediments 3—Saalian (Warta) fluvioglacial sediments; 4—Saalian (Warta) glacial sediments; 5—Vistulian (pre-LGM) fluvioglacial sediments; 6—Vistulian (post-LGM) kame terrace; 7—Holocene peat (Wierzbicki et al., 2020, modified).

The floodplain overlies an extensive peat deposit of 2–5 m which, in turn, partially overlies a 1–4 m gyttja layer. Together with a 10 m deep sandy-gravel bed, the described deposits form a single aquifer (Fig. 2). The valley intensively drains the surrounding upland and the outwash plain. It should be noticed that the present course of the Biebrza river, is not geometrically consistent with the peat system in this area (Fig. 1). It was proved that the peat forming process has been started after the last glaciation and peat deposits are filling the valley formed by melting iceberg. The river is located in the upper layer of the organic deposits (Pałczyński, 1975; Zurek, 1975; Oświt, Pacowski & Zurek, 1975). As reference values of spatial range and thickness of the peat layer for this area in numerical models, special effort was paid to the identification of this layer with different parameters.

### Conceptual model

**Figure 3:**
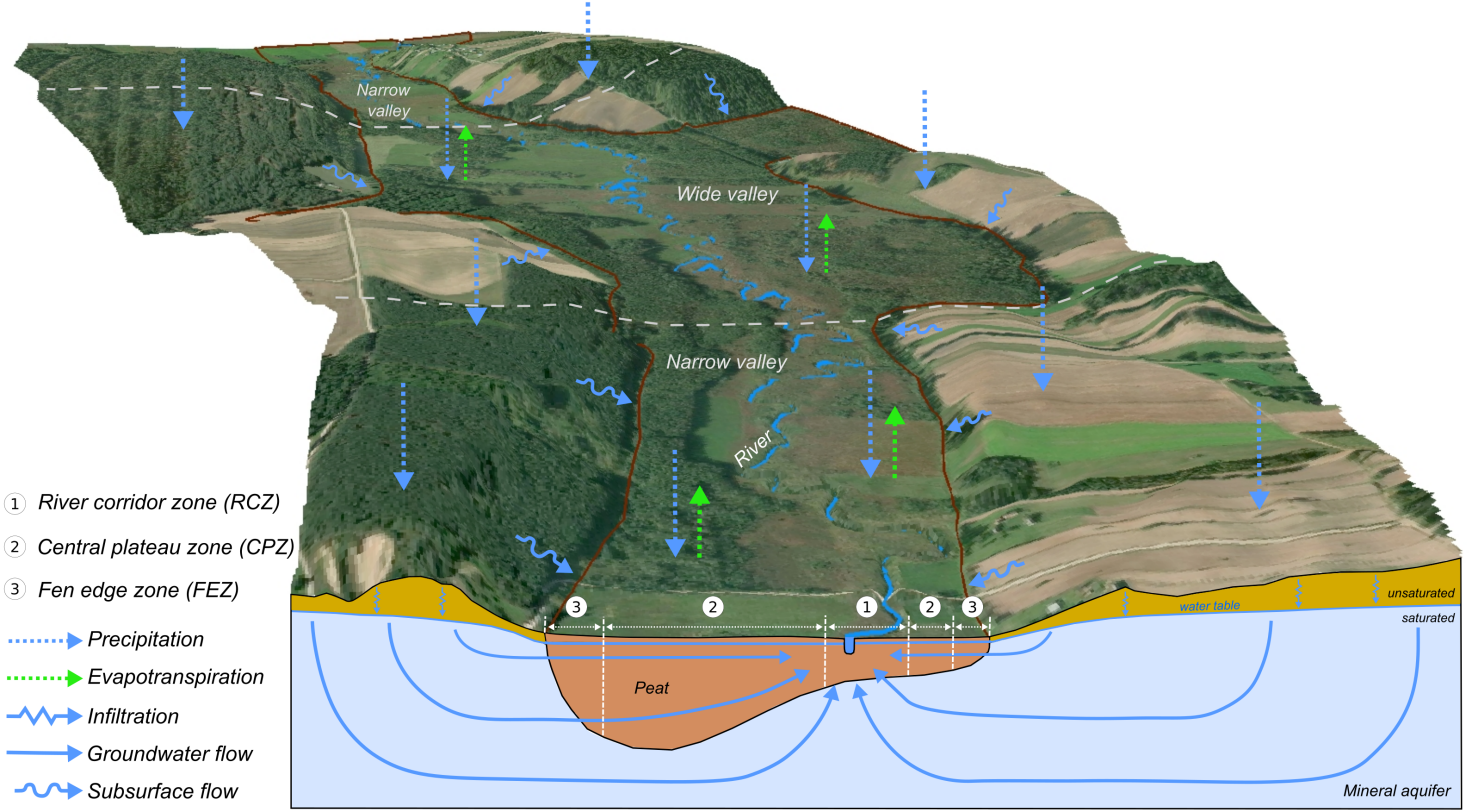
Conceptual model of water flow in the Upper Biebrza Valley.

The conceptual model considered for later balance simulations for the peat layer in the Upper Biebrza area assumes the following inflow/outflow elements: mineral aquifer, subsurface run-off from the upland (subsurface flow), river, precipitation, evapotranspiration, infiltration. These elements are schematically shown in Fig. 3. The main drainage center in this area is the Biebrza River. Only after heavy rains does it take on a temporary, infiltrating character. Drainage also occurs through evapotranspiration processes—mainly in areas where the groundwater table is located close to the surface. The peat layer is fed by the inflow from the mineral aquifer layer and by the subsurface flow from the upland. The following zones were also distinguished due to characteristic processes that occur in the aspect of water flow and river-peat interactions: the river corridor zone (RCZ), the central plateau zone (CPZ) and the fen edge zone (FEZ) depending on the distance of its occurrence from the river. The RCZ has been separated as a subregion closer to the river bank based on the analysis of Nawalany et al. (2020). The processes of direct water exchange between the peat and the river occur here. CPZ has no connection with the river bank and the edge of the valley, the main processes here are groundwater inflow from the FEZ and evapotranspiration. FEZ is connected to the edge of the valley, where we can observe a subsurface flow from the mineral part of a valley; it also has the best connection to the aquifer. Due to the different presence of zones along the valley, two types of fen valley have been distinguished: narrow (consisting of RCZ and CPZ) and wide (consisting of all three zones).

### Field measurement protocol

**Figure 4:**
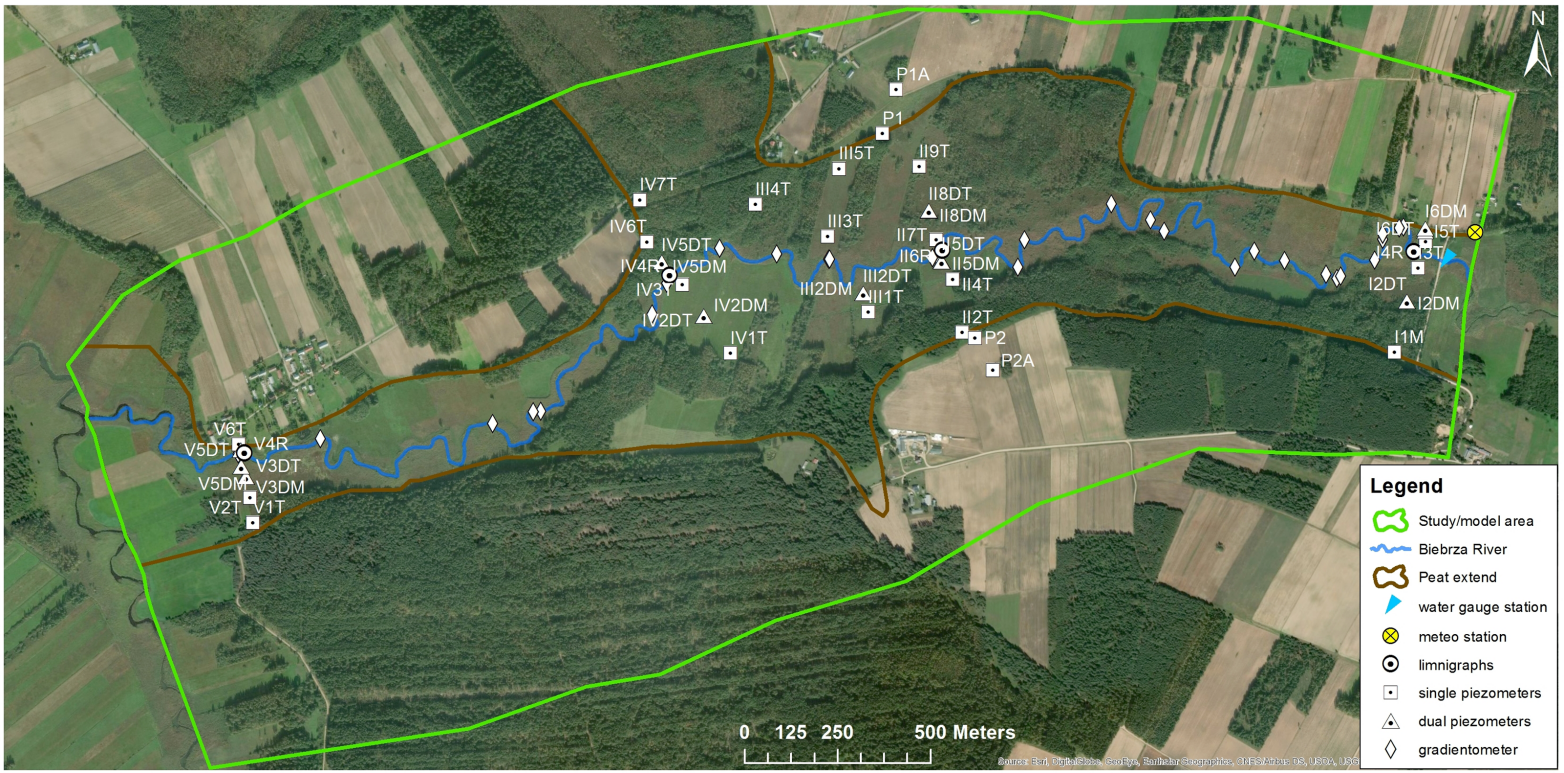
Monitoring network for the Upper Biebrza Valley—water level (dual piezometer—monitoring: water levels both in mineral and peat layers).

Taking into account the expected characteristics of the separated zones in terms of different water balance, the groundwater monitoring network was designed in such a way as to be able to verify the usefulness of these assumptions. Piezometers, where the groundwater table was measured, were distributed over the entire area, in each zone. These piezometers are arranged in lines crossing the river perpendicularly—this may allow for the detection of different zones in terms of the trajectory of water particles movement depending on the distance from the river. Groundwater tables were measured with 36 piezometers (23 shallow piezometers located in peat; nine shallow piezometers located in mineral soil and four deep piezometers reaching and monitoring the aquifer in mineral soil) equipped with pressure transducers recording water levels in 3-h intervals over the period of July 2018 to June 2019. In addition, four piezometers monitoring the water level in the river in Biebrza. In selected piezometers placed on peat and mineral soil, hydraulic conductivity values were measured by a common slug tests (rising-head tests with use of pump). The monitoring network for the Biebrza Valley is presented in Fig. 4.

As part of the monitoring, the character of the water exchange between the river and the aquifer was also investigated. These studies were carried out in July 2018, November 2018 and June 2019. The study consisted of direct measurements of the differential pressure between the hydraulic height of the river and its aquifer. The device used, called a gradientmeter, was designed to measure the pressure differences over time directed in the river. The device only measures the differences between the pressure in the aquifer and the river. When the pressure at the bottom layer is higher than the atmospheric pressure, the river, lake or reservoir is infiltrating. When the reverse is true, the water drains off. The measurement points are indicated in Fig. 4.

### Modeling

The groundwater flow model of the study area was performed using the FEFLOW approach of FEFLOW (Diersch, 2014). This software package works by solving the equations of flow, mass, and heat transport in porous and fractured media by a multidimensional finite element method for complex geometric and parametric situations including variable fluid density, variable saturation, free surface, multispecies reaction kinetics, nonisothermal flow, and multidiffusive effects (Diersch, 2014). The FEFLOW is used to model this Biebrza Valley site, in which mathematical equations are solved, as mentioned before, using the finite element method, which provides an opportunity to create a quite precise discretization grid. An initial stage in the design of groundwater model using FEFLOW is mesh generation. The 3-D groundwater model for the Biebrza River is described by the triangular mesh method, which is notable for its ability to develop complicated combinations of polygons, lines, and points at high speed. Division of the model into triangular calculation blocks enables a precise, flexible, and accurate reproduction of complicated elements of the structure of the model aquifers (river boundaries, *etc.*), guaranteeing practical and precise replication of the real shape of the studied area (Leiter, 2017).

The structure of the model includes 11 layers, 12 horizons, 1037575 elements, and 568764 nodes, which cause the complexity of the model. The aquifer structure is covered by an unconfined layer. The dimensions of the modelling (soil deposits as well as water) layers, in particular the width and thickness, were determined and estimated from the field measurement data of the piezometers as well as their lithological profiles. The parameters required for every layer were then introduced into the model, including the initial hydraulic head, flow boundaries, soil properties such as hydraulic conductivity and specific storage, precipitation data, and river assignment.

The Biebrza River is identified with the first type boundary condition (BC), that is, a fixed reference water level with measured values that change over time. This geometry was introduced into the model after analysis and interpretation of bathymetric measurements carried out in 40 cross-sections of the modeled river segment. The resistance of the bottom sediment is introduced into the model using the model layer as a parameter of the resistance of the river bed. The extent of the peat is shown in Fig. 1.

At the model boundary, the second type of BC is assumed. It is mainly a simulated subsurface run-off from the upland. The value of this inflow/outflow was the parameter that was changed during calibration.

Data on precipitation and other meteorological data were obtained from the meteorological station located in the research area (Fig. 2). In the adopted calculation method in FEFLOW, the total recharge of the aquifer is calculated by reducing the amount of infiltration by the amount of evapotranspiration. The ET0 reference evapotranspiration was calculated using an FAO Standard (Allen et al., 1998). The aeration zone was not included in FEFLOW, hence the amount of potential evapotranspiration was used for the calculations. In the ETP calculation, seasonally averaged crop coefficients based on Brandyk et al. (1996) were used. In this area, spatial—temporal differentiation of the model recharge parameters was introduced, since taking into account evapotranspiration and subsurface run-off from the upland is essential. Spatial distribution of the amount of recharge of the aquifer was adjusted in the model calibration process.

It has been estimated that the highest recharge in the model should cover the zone where the subsurface runoff from the upland is expected at its edge. Reduced evapotranspiration rate was assumed in the areas where we identified terrain depression where the surface water persists and does not flow towards the drainage center.

### Calibration and verification

To calibrate the model, measured values of the water table in piezometers were used. The model was optimized using the “trial and error” method. This method consists in manually changing the model parameters and observing the changes it causes as a result of the model. The parameters that were changed in the calibration process were the hydraulic conductivity (in the range not exceeding + −15% of the parameter value obtained from the slug test) and recharge of the model.

The calculated average error values between the observed values and those calculated by the model can be used to assess the quality of model. The model was considered a measure of compliance with the real system: (1) statistical fit indicators (Taylor, 1997) such as the MAE (mean absolute error), RMS (root mean squared) error and correlation coefficient (R); (2) the consistency of the water table over time, *e.g.*, the reactions of the model over time to changing conditions. The model was calibrated for the period from July 10, 2018 to October 31, 2018. Later data (up to June 25, 2019) were used to verify the model.

The model was also verified with independent measurements of other variables, by comparing its results with measurements of water exchange character between the river and the aquifer (gradientmeter) and hydrometric measurements carried out on September 2018 and June 2019.

## Results

### Measurements

In selected piezometers placed on peat and mineral soil, hydraulic conductivity (*k*) values were measured by a slug test. Because of the specific nature of the peat layer, which is characterized by high heterogeneity, the results of the *k* value identification should be treated as an approximate result. The more tests in different locations and depths are performed, the more accurate determination of the *k* values range is. With this in mind, the results of slug tests served as an first estimation of the representative generalizing average *k* value of the peat during the construction for the conceptual model of this area. The results are listed in Table 1.

**Figure 5:**
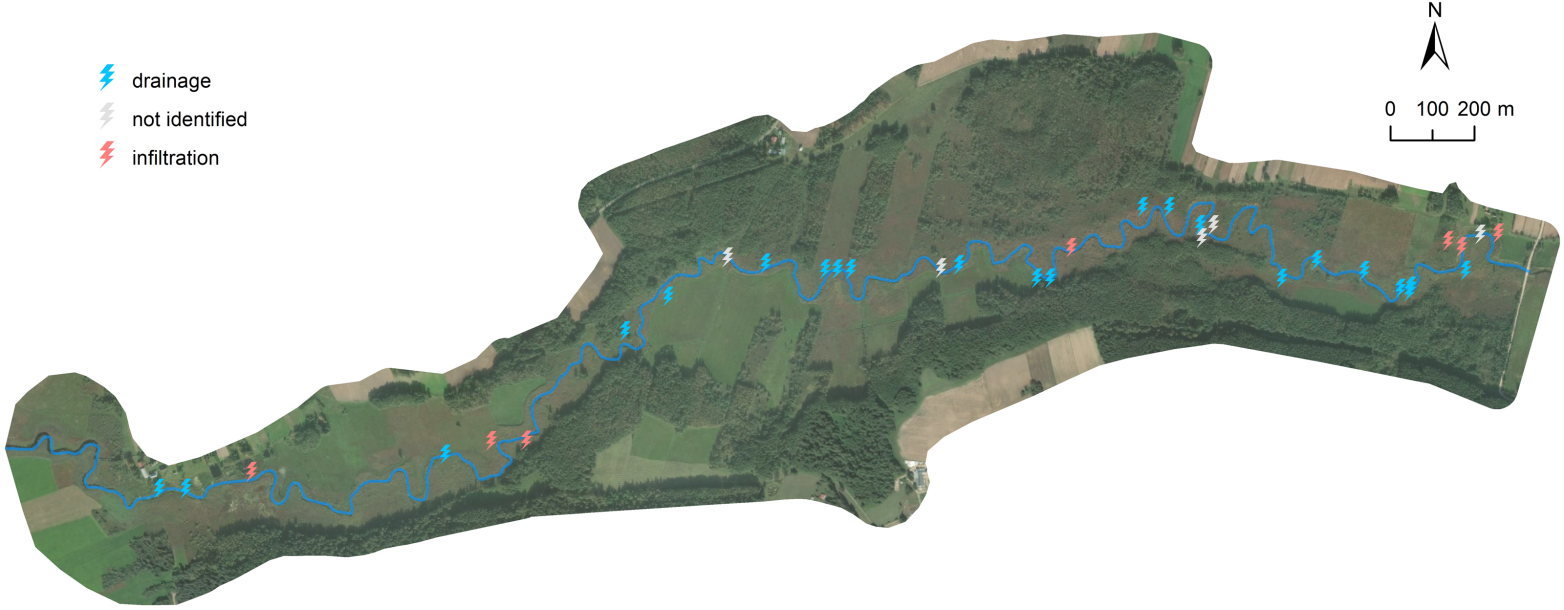
Character of water exchange between the river and the aquifer.

The nature of water exchange between the river and the aquifer was also measured along the length of the modeled river section. A gradientmeter was used to determine the hydraulic gradient between the aquifer and surface water. The results of the determination of the character of the Biebrza river are shown on the map below (Fig. 5).

### Model calibration and validation

The range of values for the hydraulic conductivity obtained by the modelling is summarized in Table 2. Peat is characterized by a high heterogeneity of its parameters. The average values of the hydraulic conductivity defined in the model are slightly different from the value of this parameter obtained as a result of slug tests. This is due to spatially the need to distribute the value of this parameter over the area of the model, having only the point information at its disposal. The point information on the hydraulic conductivity has been preserved in the model.

**Figure 6:**
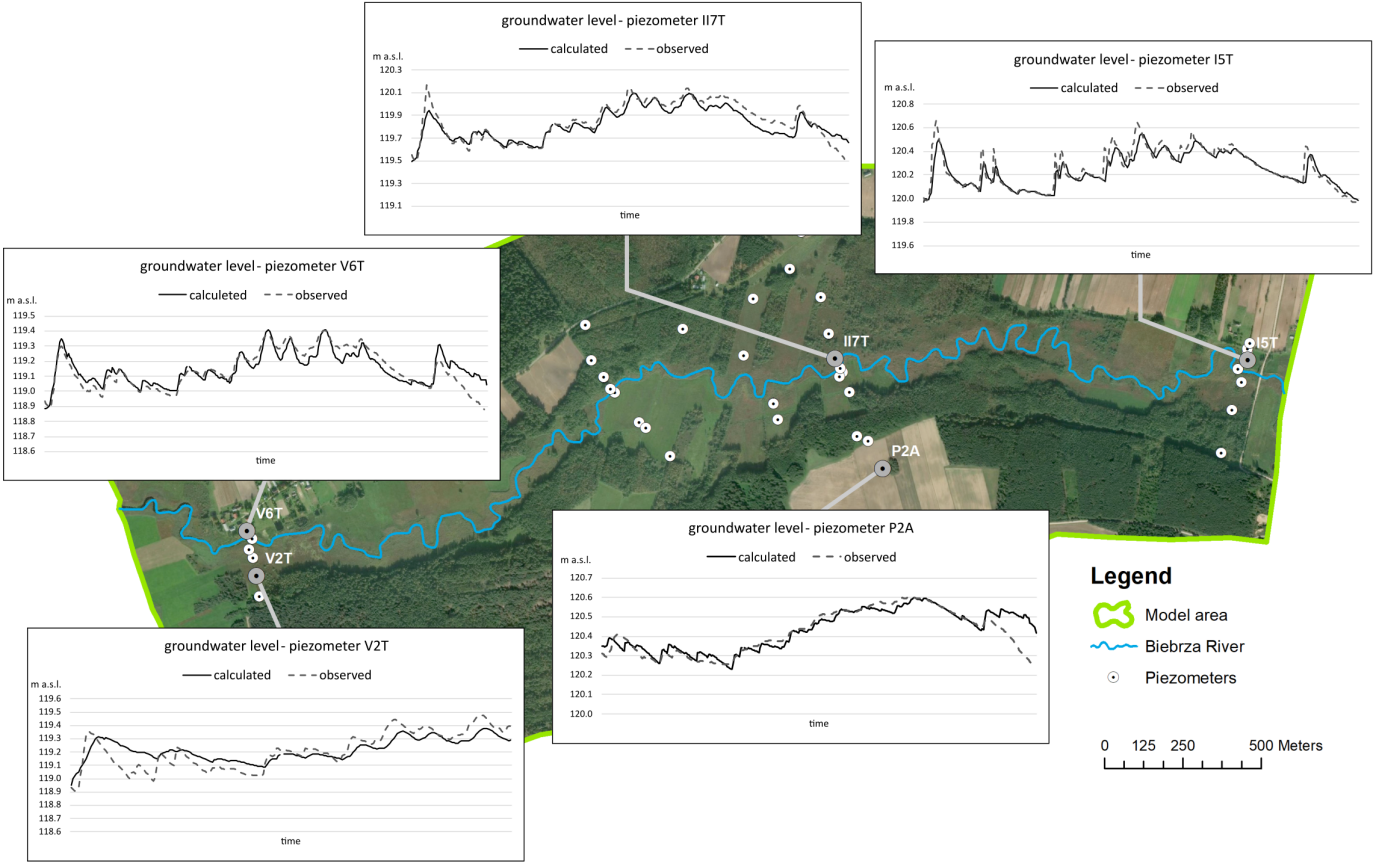
Calibration results and verification of the model.

The results of calibration and verification are shown on the graph below for selected piezometers (Fig. 6). The spatial distribution of the monitoring network with all piezometers is shown in detail in Fig. 3 and the map in Fig. 6 shows its distribution schematically. The best results of fitting the model to the measurements can be observed upstream and closest to the river. It is influenced by a very good adjusted value of the boundary condition defined in the river and sufficient identification of the filtration conditions in this area. Downstream, the boundary condition defined on the western border of the model had a large influence on the correctness of the model. The results of the calibration gave the following statistics: RMS = 0.16 m and MAE = 0.14 m. The correlation coefficient observed and calculated is 0.79 for all piezometers, taking into account 353 time steps in the model.

**Table 1:** Hydraulic conductivity values determined by the pumping test.

Hydraulic conductivity k [m/s] ×10-4									
Mineral layer					Peat layer				
Piezometer number	k	k_min_	k_max_	k_av_	Piezometer number	k	k_min_	k_max_	k_av_
I6DM	9.81	0.33	9.81	2.11	I2DT	0.92	0.04	0.92	0.30
II5DM	0.42				I6DT	0.04			
II8DM	1.58				II5DT	0.13			
III2DM	0.33				II8DT	0.23			
IV2DM	1.14				III2DT	0.19			
IV5DM	1.01				IV2DT	0.36			
V3DM	0.47				IV5DT	0.43			
					V3DT	0.14			

**Table 2:** Values of hydraulic conductivity after model calibration.

Hydraulic conductivity k [m/s] ×10-4 (after model calibration)					
Mineral layer			Peat layer		
k_min_	k_max_	k_av_	k_min_	k_max_	k_av_
0.12	3.47	2.20	0.06	2.31	1.04

The obtained value of the correlation coefficient for these specific soils meets the requirements of mapping the real hydrodynamic field. As in studies on similar aspects of the river-fen interaction (Feinstein et al., 2020) to confidence and verifying the model, a comparison of the results of river character measurements against the calculated river character was made using a mathematical model. Twenty-nine of the 34 measurements taken during the three measurement series were taken into account. In the case of five measurements, there was not enough evidence to unambiguously confirm unambiguously the nature of the river with a measuring device. The model and measurements showed convergent results considering the direction of river-peat exchange in 76% of cases (22 of 29 measurements).

### Sensitivity analysis

**Figure 7:**
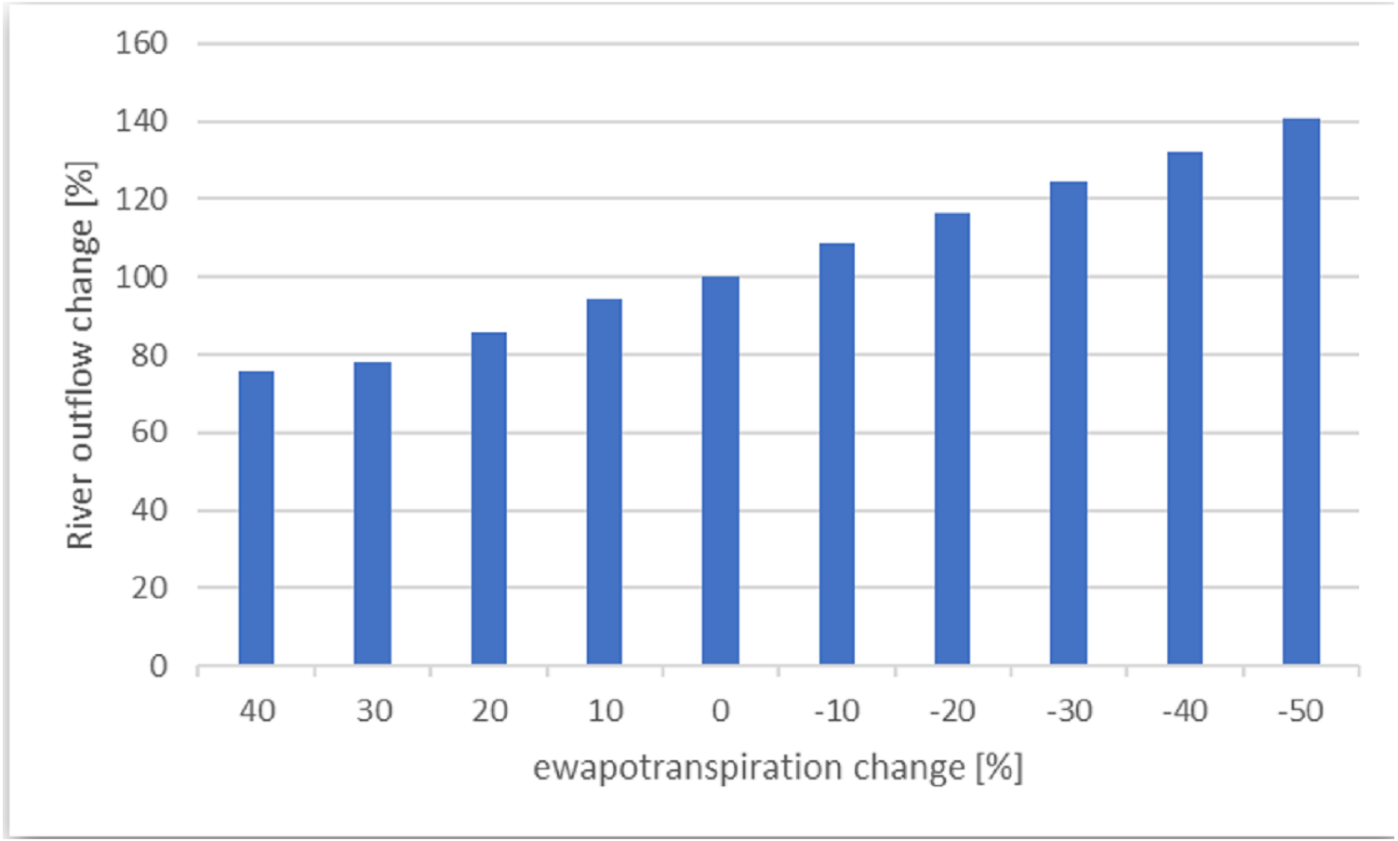
Sensitivity of river outflow to evapotranspiration.

The sensitivity of the model to changes in the following parameters and boundary conditions was analyzed: evapotranspiration, infiltration, hydraulic conductivity, and specific storage. The sensitivity of the model was analyzed in terms of the calculated water balance for the peat layer. After preliminary calculations, it turned out that the change in hydraulic conductivity and specific storage does not result in significant changes in the calculated water balance for the peat layer (the variability of water outflow to the river is within the range 10–15%). For this reason, the focus was on the influence of changes in model supply parameters on precipitation and evapotranspiration. The model is sensitive to the change in the recharge/evapotranspiration value. The error of estimating evapotranspiration can be estimated around 30% (based on Djaman et al., 2015; Grygoruk, Batelaan & Miroslaw-Swiatek, 2014)—the amount of peat layer drainage by the river will change then by 20% (Fig. 7). Decreasing the amount of precipitation that recharges the aquifer has the effect of increasing the river’s value of the water supply from the river by, at most, 40%.

### Balance of water/budget

**Figure 8:**
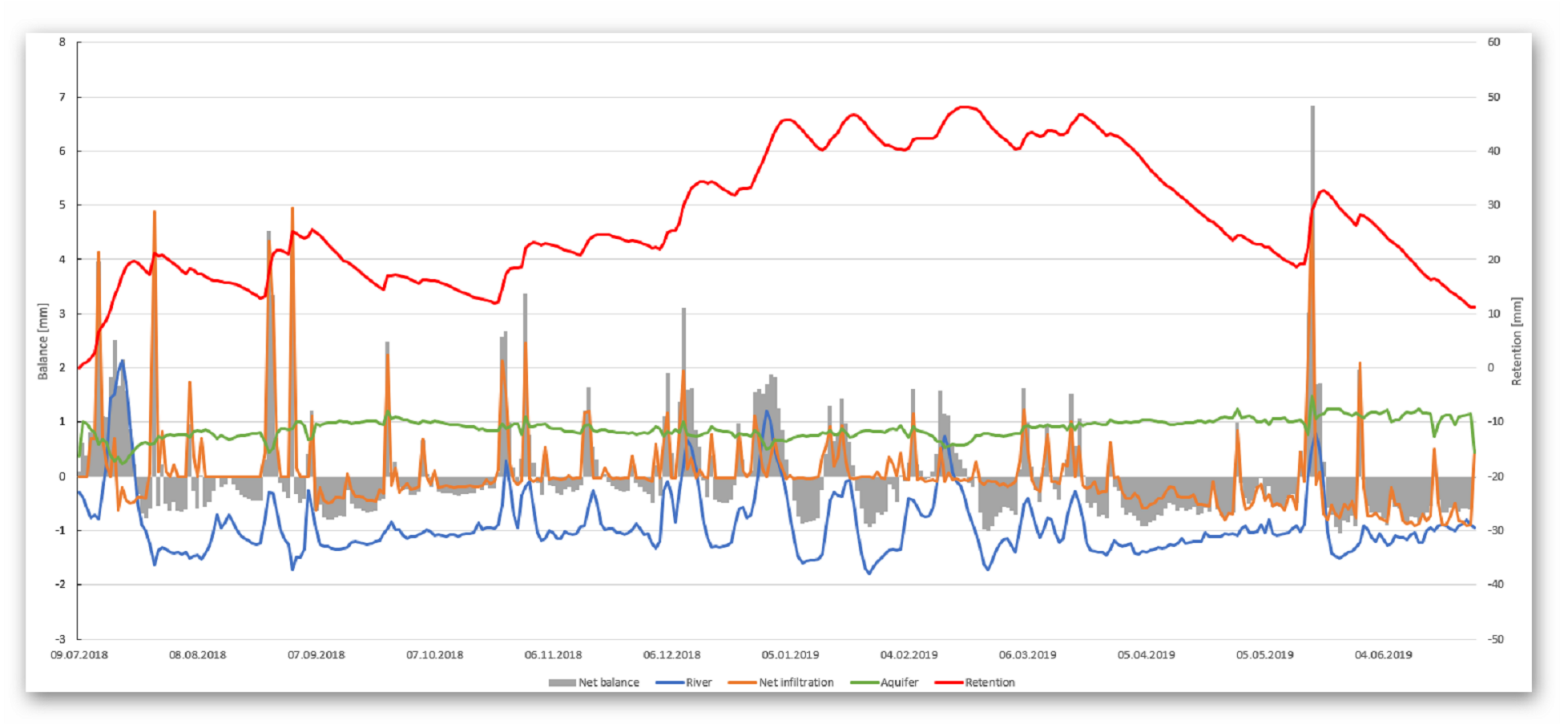
Variability of the water balance in the peat deposit.

It is clearly seen (Fig. 6 and Table 3) that the main drainage element in the model area is the river (about 82% out). Evapotranspiration is around 17% and occurs mainly in peat. Less than 1% of the water flow is the amount of water that flows out through the west boundary of the model. Considering the recharging of the model, it can be noticed that the two sources, infiltration and inflow from the valley, are similar to the amount of water. Particularly noteworthy is the analysis of water exchange between the peat and other considerable elements of the water balance: the river, peat deposit, and mineral aquifer. Analysis of the graph shown in Fig. 8, which takes into account the peat layer, clearly shows clearly that the peat is recharged mainly from the horizontal mineral soil layer. This recharging is kept constant. Peat layer recharging from other sources (river, net infiltration) looks rather incidental and is only visible during precipitation, but it does have a meaningful value when considering the amount of water: river approx. 5% and infiltration approx. 15%. Taking into account the net balance, it can be stated with certainty that the main drainage in this area is the river (about 64% out). Consequently, 24% and 12% of the total flow flows into the mineral soil and through evapotranspiration. In general, the net balance in peat in the modelling time is positive (around 10 mm).

**Table 3:** Comparison of the water balance domain and peat deposit.

	Whole model domain						Distinguished peat deposit				
	IN		OUT		IN-OUT		IN		OUT		IN-OUT
	[m^3^]	%	[m^3^]	%	[m^3^]		[m^3^]	%	[m^3^]	%	[m^3^]
River	52646	5.7	710907	82.0	−658261	River	52646	4.6	710907	63.9	−658261
Net infiltration	447674	48.3	147894	17.1	299779	Net infiltration	164647	14.5	137939	12.4	26707
Upland	425715	46.0	7638	0.9	418077	Mineral aquifer	918 698	80.9	264066	23.7	654631
					Including subsurface flow	62 303	5.5			

**Figure 9:**
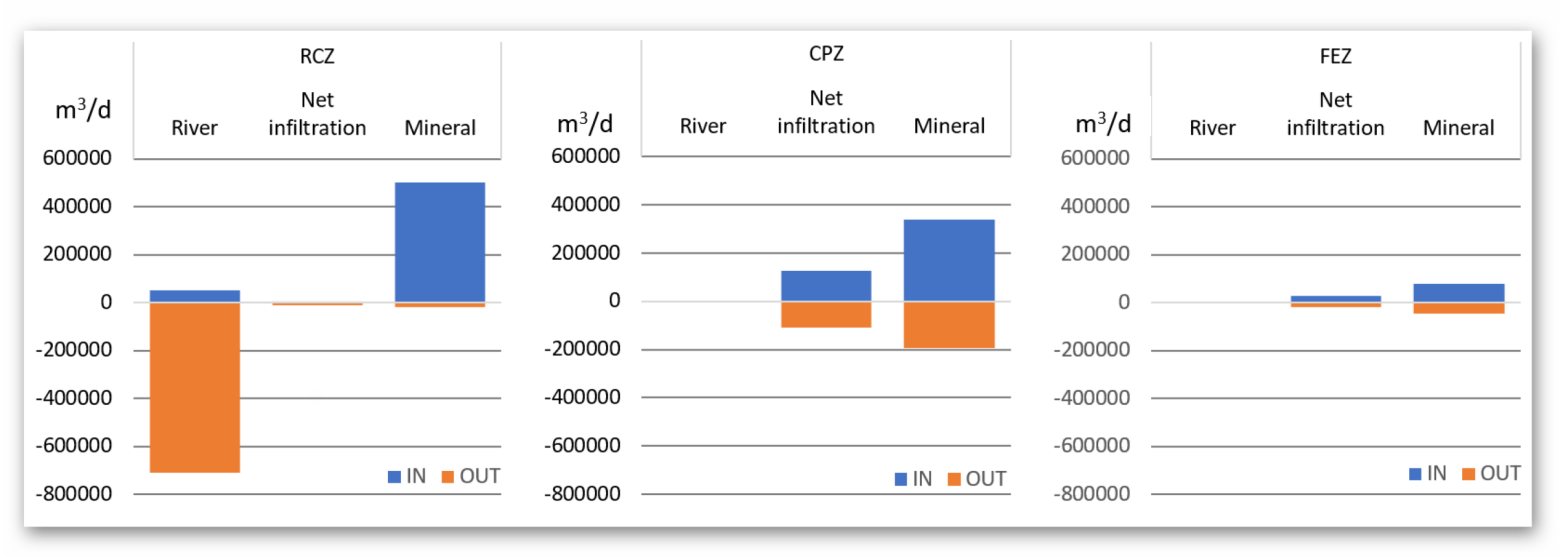
Summary of water balance in peat valley zones.

The results of water balance has confirmed our concept on distinguishing different elements of the fen valley elements on river corridor zone (RCZ), the central plateau zone (CPZ) and the fen edge zone (FEZ), which differ in intensity of hydrological processes intensity. Figure 9 presents a summary of the water balance in the proposed fen river valley zones. It can be certainly stated that the RCZ is characterized by water outflow mainly through the drainage river. The flow of water in this zone is forced by the pressure difference between the river and the peat layer. Water flows into this zone mainly from the infiltration of the aquifer and the lateral inflow from the surrounding peat zone. In the central plateau zone, a significant share of evapotranspiration processes can be observed in the outflow of water from the system. In the fen edge zone, the water flows horizontally towards the river, feeding the water to the central peat zone.

**Figure 10:**
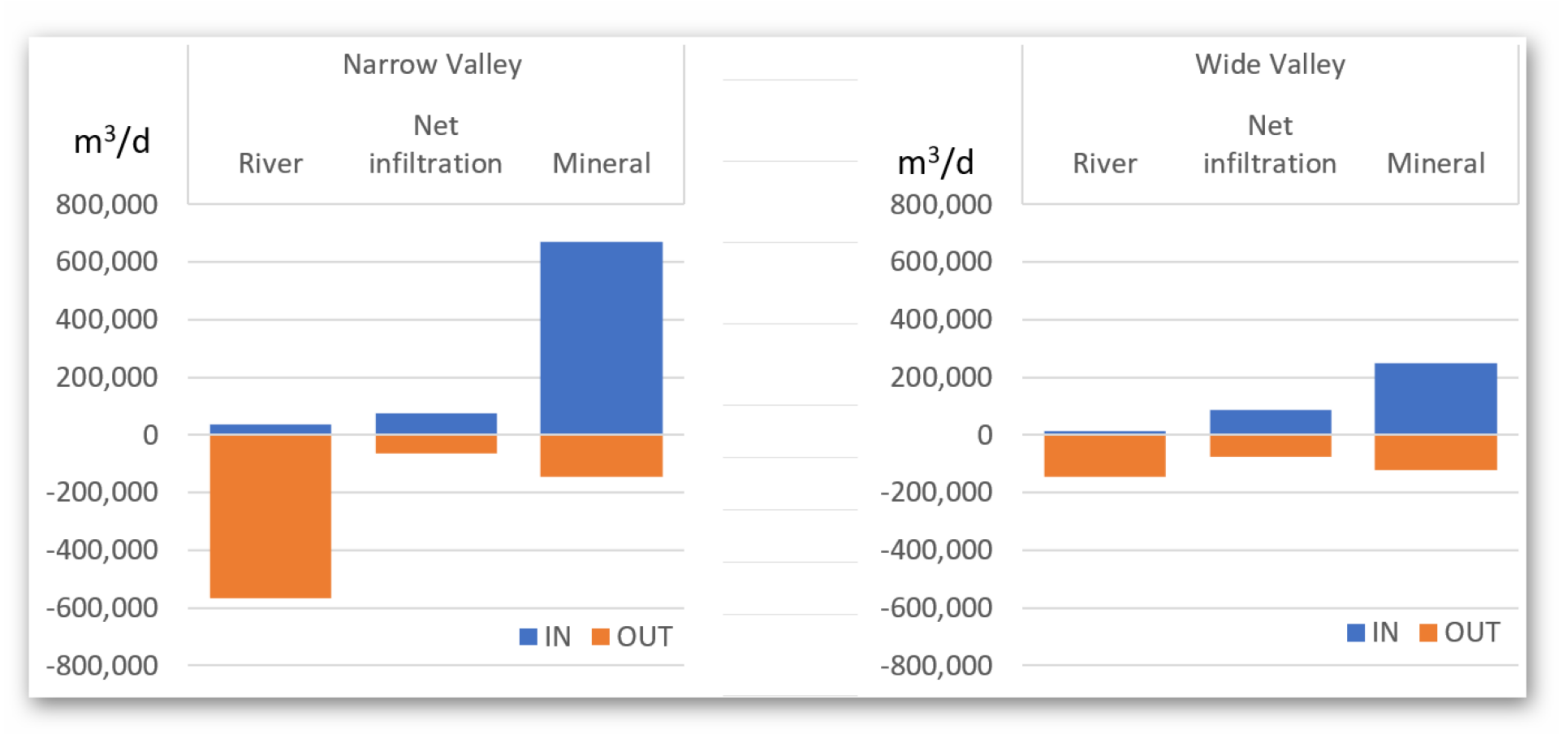
Summary of the water balance in designated valley types.

To check how the width of the river valley affects the characterized water exchange rates, the distribution of water exchange in the zone with a narrow and wide valley was also checked and summarized in Table 4 and Fig. 10. The division of the valley into narrow and wide is presented in Fig. 3.

This analysis confirms that for the narrow river valley the contribution of the river in the outflow of water from the system is significantly higher than in the case of the wide valley. This impacts the conservation strategy, where special attention should be paid to the river status (canal depth, bottom sediments presence, average water level, *etc*). In a wide valley, the river does not receive as much water from the peat system—here most of the water outflows to mineral aquifer and through evapotranspiration drainage. In the case of the Biebrza River, the narrow valley is 200–300 m wide, while the wide valley is wider, with a maximum of 600 m.

**Table 4:** Summarized model balance for the narrow and wide valley.

	Narrow valley						Wide valley				
	IN		OUT		IN-OUT		IN		OUT		IN-OUT
	[m^3^]	%	[m^3^]	%	[m^3^]		[m^3^]	%	[m^3^]	%	[m^3^]
River	37639	4.8	565063	73.1	−527424	River	14898	4.2	144273	42.1	−129374
Net infiltration	75695	9.6	64896	8.4	10799	Net infiltration	88950	25.2	75261	22.0	13689
Mineral layer	670711	85.5	143552	18.5	527159	Mineral layer	249101	70.6	123049	35.9	126051
Including subsurface flow	30123	3.84			Including subsurface flow	7079	2.0			

## Discussion

The groundwater monitoring network in the study area was designed to verify the conceptual model in terms of the usefulness of distinguishing the three RCZ, CPZ and FEZ zones. Both the groundwater head changes and the amount of precipitation were measured. The measurement points were located in different zones throughout the analysis area, which allowed to study the spatial variability of the water flow characteristics and water balance over the entire area. The applied 3-hour measurement interval between measurements makes it possible to detect differences in the water balance immediately after the occurrence of sudden changes in conditions, *e.g.*, rainfall. The problem, however, is that these data were collected in the period June 2018–June 2019, which was a dry period. The annual sum of rainfall is between 0.5 and 0.6 percentiles determined for the multiyear period 1961–2019, which would indicate average conditions (NOAA, 2022). For this reason, this model is not calibrated with sufficient accuracy for wet-period utility. Generally, the monitoring network was found to be useful for model calculations.

To calibrate the model, the measured values of the water table in piezometers were used. The results of the models were compared with them, taking into account the general calibration statistics for stationary models (MAE, RMS, R) and the reactions of the model over time to changing conditions. The obtained values of hydraulic conductivity are representative of the conceptual values suggested by previous research on the peat and mineral layers presented in the paper by Anibas et al. (2012). It is also in accordance with the hydraulic properties of peat soils presented by the literature review in (Liu & Lennartz, 2019) who set together 24 published studies on peat properties. Nevertheless in the literature (*i.e.,* overview in Kaczmarek, Dabska & Popielski, 2022) pointed out that *k* value can be much smaller (especially in Polish conditions). Such situation is related to the used method (field tests usually give the higher *k* values then in case of lab testes interpretations) as well as the very low stress state affecting soil skeleton (cause of low bulk density of peat, no additional loading, near surface location, buoyancy of water).

The correct mapping of the modeled groundwater system was also verified by measurements: the hydrometric character of the river was measured and compared. In the case of the verification of model results with hydrometric measurements, the same order of magnitude of river-peat water exchange was obtained both in total and by comparing the results between river piezometers. In 76% of the cases of studies on the nature of the Biebrza River in terms of interaction with the peat layer, satisfactory agreement was obtained with the model. For the vast majority of measuring points, where the drainage character of the river was demonstrated, the model also showed the same character. For measuring points where the river is infiltrating, the model does not show such a character. The river assumes an infiltrating nature immediately after precipitation; no measurements were taken in such periods. Checking if the measurement method is effective would require carrying out a series immediately after rainfall.

Due to the high sensitivity of the model to changes in evapotranspiration values, the possible effect on the balance of the peat layer in the model was analyzed. The error of estimating evapotranspiration can be estimated around 30% (based on Djaman et al., 2015; Grygoruk, Batelaan & Miroslaw-Swiatek, 2014)—after calculation of the balance for the peat layer, the balance did not change in terms of the contribution of particular elements in the overall balance. The dominant contribution of the river to the outflow from the peat layer remained (65%); the contribution of evapotranspiration to the outflow from the peat layer increased to 34%. Therefore, it can be concluded that the error in estimating evapotranspiration does not affect the final result of the model balance in terms of the quantitative division of its individual components. The presented model does not calculate the aeration zone in terms of water flow. For more accurate calculations of the flow of water flow in the aeration zone, it would be reasonable to use a more accurate scale and a different model that would take this zone into account. Then the error in estimating the evapotranspiration would be reduced.

After analysis of the model results for the studies of the water balance it can be seen that the main source of peat supply in the area studied area is the aquifer (about 80%). We agree with the thesis that they are especially important in the summer half-year, as they may replenish the temporary shortages in soil moisture caused by evapotranspiration, while they are not the main source of water. Since the mineral aquifer seems to be the most important source of water for peat, one should focus on maintaining its high water content. Van Loon et al. (2009b) pointed out that elements such as drains or ditches (Schot, Dekker & Poot, 2004; Van Loon et al., 2009b) and also groundwater intakes (Fojt, 1994) intercept groundwater that is potentially directed to the fen surface and prevent available groundwater from entering the fens The conceptual division into RCZ, CPZ and FEZ zones found its confirmation in the diversified water balance calculated on the basis of mathematical modeling. In these zones, the main sources of water supply differ significantly (for the RCZ and CPZ zones it is mainly mineral aquifer). In the case of drainage sources, the largest contribution of the river in the RCZ zone and the increased amount of evapotranspiration from the CPZ zone can be seen. There are no reports in the scientific literature about this kind of diversification of water balances in the zones by rivers flowing on peats.

Diversity was also demonstrated by comparing the change in water balance changing with time for different peat zones with different spatial characteristics. In a relatively small area, three zones have been distinguished, which differ significantly in the characteristics of their water supply. The amount of river discharge water is significantly higher for the narrow valley than for the wide one. This statement is in line with previous research presented by Lobanova et al. (2018).

## Conclusions

After the analysis and interpretation of the fieldwork and modelling studies, the answers to the questions posted in the introduction to the article were formulated. The research allowed for the formulation of the following conclusions: (1) The Biebrza River has a draining character, incidentally infiltrating after precipitation. There is no significant contribution to the recharge of the peatland layer (approx. 10%). The drainage efficiency of the river may be crucial for the protection of mires or peatlands. The characteristics of this section of the river, in particular in terms of the change of spatial and temporal water exchange between the river and the aquifer, may have great consequences in determining the impact of this exchange on the peat habitat. (2) Following analysis of the model results concerning the peat layer water balance studies, it can be seen that 80% of water budget comes from groundwater. This agrees with theory and stresses the importance of aquifer protection for fens conservation. Recharging of the peat layer from other sources (river, net infiltration) looks rather incidental and is only visible during precipitation. When analyzing the drainage of the peat layer, it can be noticed that it is significantly dependent on the characteristics of the valley. In the case of wide valleys (min 300 m wide), the process of evapotranspiration plays a key role. In the case of valleys of a lower width, the river plays the most important role in draining water from the peat. (3) Morphological settings have a crucial influence on the river-fen relationship. The amount of river discharge water is significantly higher for the narrow valley than for the wide one. The influence of river drainage is extremely important for narrow valleys and there it is crucial to protect the high level of the river to limit water runoff from the fen. For wide valleys (min 300 m width), the key is to limit water runoff through evapotranspiration—here the river runoff is quantitatively smaller. The introduced three zones system: RCZ, CPZ and FEZ for river valleys with peat accumulation has been proofed as different in terms of water balance. Due to the undeniable need to introduce increased protection and restoration of marginal fens, it seems to be most important to focus on (a) the limitation of drainage of the mineral aquifer that recharges fens (b) the increased water in the peat layer by decreasing the intensity of evapotranspiration.

Further research on the intensity of water exchange between the Biebrza and peat should be carried out; differences can be seen both in the intensity of water exchange over time and in the river course. The analysis of the results so far shows that it should be done under different conditions—*e.g.*, immediately after intensive precipitation.

##  Supplemental Information

10.7717/peerj.13418/supp-1 Supplemental Information 1 Raw data collected from the piezometers

## Additional Information and Declarations

### Data Availability

The following information was supplied regarding data availability: The raw data collected from the piezometers is available in the Supplemental File.
